# Carbon-based two-dimensional nanomaterials (CB-2DMs) for high-performance wound healing platforms bridging mechanics and medicine

**DOI:** 10.1186/s11671-026-05028-0

**Published:** 2026-10-16

**Authors:** Rishabh Anand Omar, Kulwinder Singh, Neetu Talreja, Divya Chauhan, Mangalaraja Ramalinga Viswanathan, Werayut Srituravanich, Mohammad Ashfaq

**Affiliations:** 1 Faculty of Allied and Healthcare Sciences, Rayat Bahra Professional University, Hoshiarpur, 146101 Punjab India; 2 Department of Physics, Graphic Era Deemed to be University, Dehradun, Uttarakhand, 248002 India; 3 Department of Science, Faculty of Science and Technology, Alliance University, Bengaluru, 562106 Karnataka India; 4 Department of Drinking Water and Sanitation, Ministry of Jal Shakti, 1208- A, Pandit CGO Complex, Lodhi Road, New Delhi, 110003 India; 5 Faculty of Engineering and Architecture, Universidad Arturo Prat, Avenida Arturo Prat 2120, Iquique, 1110939 Chile; 6 Department of Mechanical Engineering, Faculty of Engineering, Chulalongkorn University, Pathumwan, Bangkok, 10330 Thailand; 7 Research & Development Cells, and School of Sciences, Woxsen University, Hyderabad, 502345 Telangana India

**Keywords:** Wound healing, Dressing materials, Polymers, 2DMs, Mechanical strength

## Abstract

Wound healing is a highly complex biological process encompassing hemostasis, cellular proliferation, inflammation, and tissue remodelling, which are strongly influenced by the mechanical microenvironment of the wound site. Wound healing remains a critical clinical challenge due to the constraints of traditional dressing materials, which often lack sufficient mechanical strength, flexibility, and adaptability to wound microenvironments. In this context, carbon-based two-dimensional nanomaterials (CB-2DMs) such as graphene, graphene oxide (GO), graphitic carbon nitride (g-C_3_N_4_), and carbide-MXene have emerged as promising candidates for smart wound-dressing materials due to their remarkable mechanical strength, flexibility, and tunable physicochemical characteristics. Moreover, CB-2DMs incorporated into polymers make them exceptional candidates for smart wound-dressing materials, which can effectively mimic skin mechanics and maintain structural integrity under physiological stress. This review article focuses on CB-2DMs-based wound dressing materials and their mechanical characteristics, such as tensile strength, stiffness, elasticity, and durability. Additionally, we discuss the synergy between mechanical characteristics and biological functionality, such as wound healing rate and antibacterial activity. The review also discusses the current challenges and offers a promising platform for engineering CB-2DMs-based wound dressing materials to overcome these challenges and promote efficient wound healing, and how the mechanical characteristics enhance performance, stability, and clinical translation ability.

## Introduction

Wounds are described as destructive to the integrity of tissue, and the treatment/healing of wounds is one of the greatest challenges nowadays. The healing of wounds is achieved through four processes, including hemostasis, inflammation, cell proliferation, and tissue remodelling. Although the healing processes of wounds are normal, severe wounds like burn wounds damage the primary defence barriers (epithelium and endothelium layers) of the skin [1–4]. Severe wound infections combined with significant fluid loss and dehydration can rapidly progress to systemic complications and become life-threatening, potentially leading to death if left untreated [2, 3, 5–9]. In this aspect, there is a necessity for innovative wound dressing materials that might speed up the healing process as well as control the loss of blood and bacterial infection.

Traditional dressing materials lack water-retention ability and are inefficient in controlling bacterial infection. The ideal wound dressing materials should have high air permeability, high sorption ability to absorb exudate from wounds, and augment the healing process of wounds. The researchers continue looking towards newer dressing materials, which promote moist environments and enhance healing processes [10–15]. Numerous materials, including carbon-based nanomaterials (CBNMs) such as carbon nanotubes (CNTs), carbon nanofibers (CNFs), graphene, carbon dots, and their composites, have been used to develop wound dressing materials [16–20]. Among them, carbon-based two-dimensional nanomaterials (CB-2DMs) such as graphene, graphene oxide (GO), reduced GO (r-GO), graphitic carbon nitride (g-C_3_N_4_), and MXene have gained significant interest from researchers due to their exceptional characteristics, such as high surface area, mechanical strength, and biocompatibility. Mechanical properties are among the most important characteristics, as they affect various properties of the materials, mainly elasticity and elongation. These characteristics are among the most important for wound dressing materials [21–29]. Interestingly, the insignificant toxicity of the CB-2DMs against various cell lines makes them suitable for wound dressing materials either directly or indirectly (as fillers).

**Fig. 1:**
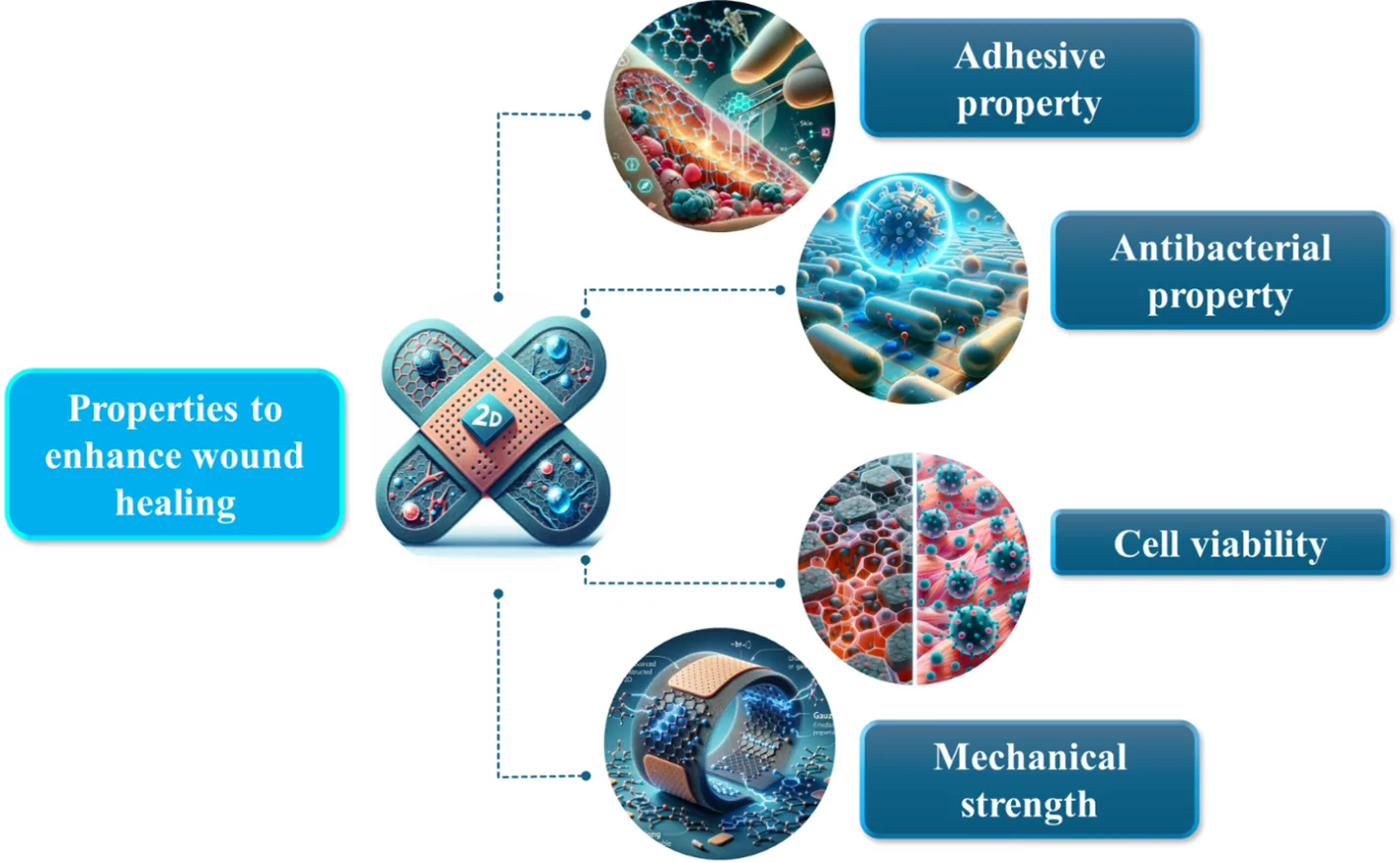
A schematic illustration of the CB-2DMs-based wound dressing materials and their characteristics. This artwork was produced using Microsoft Copilot Studio

Recently, several CB-2DMs such as graphene, GO, r-GO, g-C_3_N_4_, and MXenes have been synthesised for numerous applications, including wound dressing materials, due to their properties such as high surface area, large pore volume, high mechanical and electrical properties, and excellent biocompatibility [30–32]. The outstanding strength of graphene and its derivatives is one of the reasons that make graphene stand out both as an individual material and as a reinforcing agent in composites. The reason for the exceptional mechanical properties of graphene lies in the stability of the sp2 bonds that form the hexagonal lattice and oppose a variety of in-plane deformations [33–35]. Besides the other CB-2DMs characteristics, MXenes have a highly negatively charged surface due to surface-terminating groups, mainly hydroxyl (-OH), oxygen (-O), and fluorine (-F) incorporated during their synthesis, thereby easily mixing with other materials, including polymers [36, 37]. Numerous polymers such as chitosan, poly-vinyl alcohol (PVA), hyaluronic acid (HA), cellulose, gelatin, silk fibroin, and agarose have been used for wound dressing materials, as they maintain moist environments and protect against the outer environment. Moreover, such polymers have film-forming ability, so that easily be applied anywhere on the body [38–42]. Furthermore, incorporating CB-2DMs within the polymeric matrix significantly enhances mechanical strength and flexibility, making them suitable candidates for wound dressing materials. Indeed, the uniform distribution of the CB-2DMs within the polymeric matrix is of utmost importance, which affect the performance of wound dressing materials. Poor dispersion/agglomeration may adversely affect the mechanical properties; therefore, homogeneous distribution of CB-2DMs is essential for maximising their reinforcing effects. The mechanical strength of the CB-2DMs is one of the significant features, which has several advantages for wound healing applications, especially for designing dressing materials. High tensile strength is an important feature of any wound dressing material, as high-tensile dressing materials can be easily wrapped around any part of the body [43–47]. Undoubtedly, there are plenty of reasons to consider CB-2DMs in wound dressing materials, especially the mechanical properties that affect the growth of cells and control bacterial infection. Figure 1 shows the schematic illustration of the CB-2DMs-based wound dressing materials and their characteristics. In this aspect, these CB-2DMs are used as fillers in different wound dressing materials to enhance mechanical properties, as well as the adhesion of cells.

Although most of the reviews mainly emphasised the characteristics of MXene, graphene, and other 2DMs, such as material synthesis, physicochemical properties, antimicrobial activity, drug delivery, and wound-healing applications. The mechanobiological aspects of CB-2DMs in wound healing, including their influence on mechanical cues, cell-material interactions, and mechanotransduction, remain insufficiently discussed. This article focuses on CB-2DMs-based wound dressing materials and their benefits, including antibacterial activity and wound healing. Moreover, this article highlights the critical role of the mechanical characteristics of CB-2DMs-based wound dressing materials and establishes a direct relationship between CB-2DMs, mechanical performance, and wound healing efficiency. The CB-2DMs show a new paradigm shift in the design of a high-performance wound healing platform and discuss the challenges related to biocompatibility, scalability, and clinical translation.

## Influence of mechanical properties on wound dressing materials

The mechanical characteristics of wound dressing materials, such as tensile strength, stiffness, elastic modulus, and viscoelasticity, etc., are of utmost importance, as dressing materials cover the wounds and act as interim mechanical barriers to inhibit bacterial growth and avoid loss of water. Clinical practices show that higher tension in the stitches during surgical procedures increases the possibility of the formation of a scar. Moreover, stress and stiffness of the wound significantly affect the rate of the healing process. However, the relationship between the healing process and mechanical properties is still unclear [48–57]. In this aspect, researchers try to understand the exact mechanism of wound healing with special emphasis on mechanical properties.

The mechanical properties of wound dressing materials, mainly stiffness and elasticity, significantly influence wound healing by regulating cell adhesion, migration, proliferation, and differentiation through mechano-transduction pathways [58]. CB-2DMs possess exceptional mechanical strength and large surface area, enabling them to reinforce polymeric dressings without compromising flexibility. The CB-2DMs provide mechanical cues that promote mechano-transduction, thereby enhancing fibroblast and keratinocyte activity, angiogenesis, and tissue regeneration. Therefore, CB-2DMs not only improve the structural stability of wound dressings but also create a favourable biomechanical microenvironment that accelerates wound healing [59, 60]. The mechanical properties are shown to be key components that could manage cellular characteristics. The mechanical properties of wound dressing materials should be tailored according to the wound types and physiological condition. For superficial wounds, a tensile strength of approximately 0.05 to 0.3 MPa is usually acceptable, whereas dressing materials proposed for high-mobility/load-bearing areas might require values of 0.3 to 2 MPa. The elastic (Young’s) modulus ideally should be similar to that of native skin to minimise stress disparity, typically ranging from 0.01 to 1 MPa depending on skin [61–63]. Recently, a few studies have been done that focus on how the mechanical properties affect cell proliferation. For instance, Engler et al. grew mesenchymal stem cells (MSCs) on the polyacrylamide (PAM) hydrogel matrix with tunable stiffness. Interestingly, MSCs show changes in differentiation patterns upon varying the stiffness from 0.1 to 2.5 kPa [64]. Another study shows that the modular PAM hydrogel exhibits mechanical memory effects using primary and secondary extracellular matrix. It was observed that the monolayer cells migrated faster in a stiff matrix and were more coordinated on the soft ECM matrix. These data indicate that the migrating cells might be able to remember from the past environment, which considerably affects cellular fates [65]. A few studies focused on the mechanical properties that affect scar formation, which show that the mechanical properties can control the growth of the cells and tissue [66–68]. It is important to discuss here that mechanical signals might modulate immune response by changing the behaviour of cells, which relates to the development of bacterial resistance in wounds [69]. These studies suggest the importance of mechanical properties in the growth of cells and tissue. Moreover, the mechanical memory of the cells shows a remarkable ability in cellular adhesion and migration, as cells can remember information from previous physical environments. Furthermore, mechanical properties affect the formation of scars and control bacterial resistance at the site of infection.

The CB-2DMs enhance the mechanical strength and flexibility of wound dressings, while providing topographical cues, which promote extracellular matrix remodelling, angiogenesis, and tissue regeneration. It is important to mention that optimum loading and uniform dispersion of the CB-2DMs might produce an interconnected reinforcing network, which promotes more effective stress distribution, thereby significantly improving modulus, strength, and structural stability. With the help of these studies, we can say that the mechanical properties of wound dressing materials might control infection indirectly by maintaining dressing structure, barrier function, and resistance to external bacterial penetration, rather than by inhibiting bacterial growth directly. The direct antimicrobial activity is attributed to the specific antimicrobial components and surface/chemical properties of the CB-2DMs-based materials. The wound dressing materials create a strong barrier to prevent the entry of bacteria and minimise the risk of infections. Additionally, some of the wound dressing materials have inherent antibacterial ability, and these materials kill/inhibit bacterial growth at the site of infection. Therefore, improving mechanical properties in the wound dressing materials might be advantageous to create an environment that helps to speed up the healing process and prevent the growth of bacteria.

## CB-2DMs as wound dressing materials

At present, CB-2DMs such as graphene, GO, g-C_3_N_4_, and MXenes have gained significant interest from researchers due to their exceptional properties, mainly high surface area, easy functionalization, and exceptional mechanical properties. Moreover, the high biocompatibility of the CB-2DMs makes them suitable candidates for various biological applications, including wound healing. Additionally, these CB-2DMs show antibacterial activity against various bacterial strains [24, 70–74]. Although the incorporation of metals and polymers appreciably improved their applicability towards wound dressing materials by increasing antibacterial activity, mechanical strength, and biocompatibility, thereby, CB-2DMs-based wound dressing materials might provide an effective solution to treat bacterial infection as well as speed up the healing process of wounds.

### Graphene and its derivatives-based wound dressing materials

**Fig. 2:**
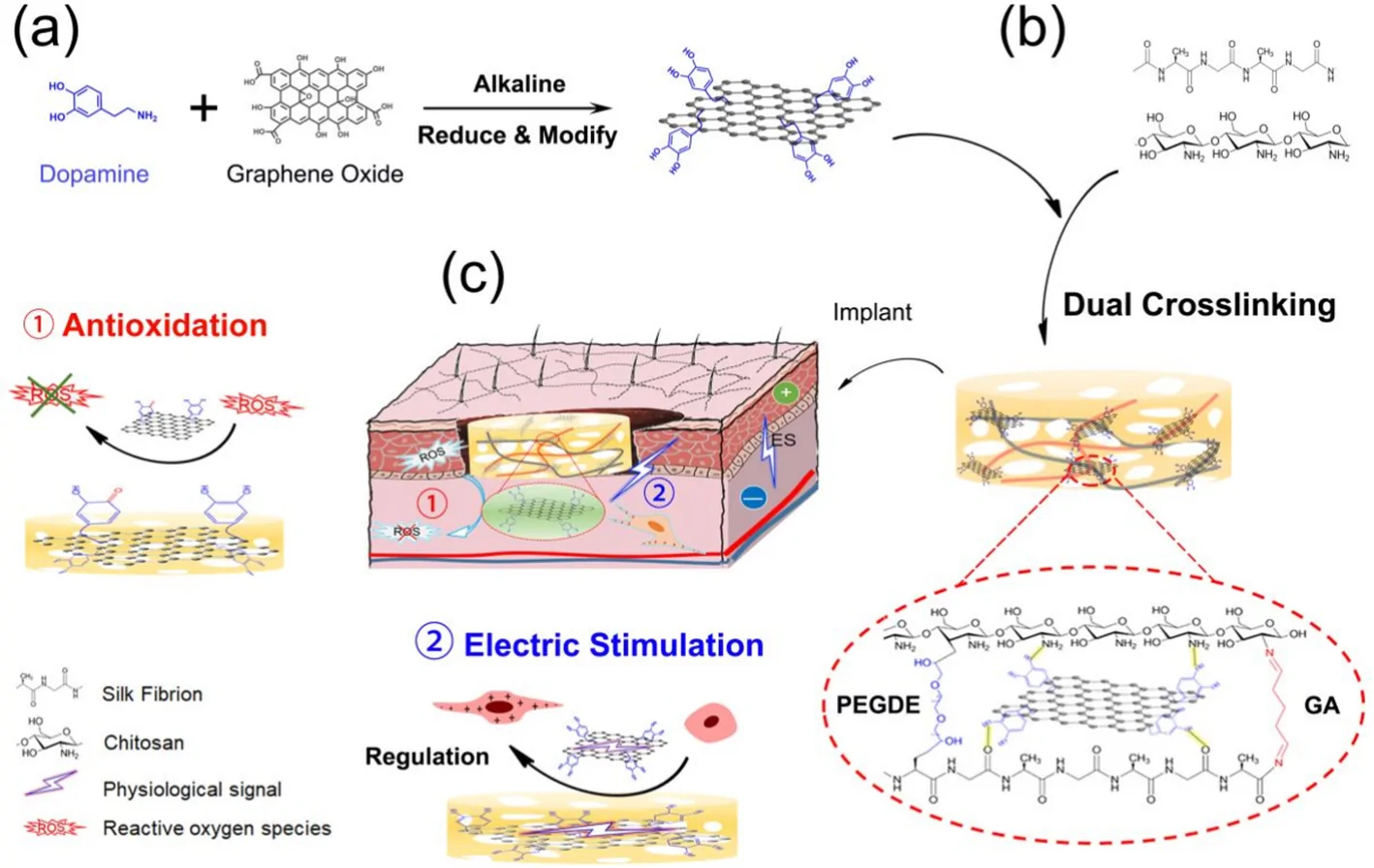
A schematic illustration of the synthesis of PGO-CSF-based scaffold for wound healing. **a** Synthesis of polydopamine-incorporated rGO, **b** Dual crosslinking of polydopamine-incorporated rGO with chitosan and silk fibroin, and (**c**) PGO-CSF-based scaffold stimulated by an electrical signal and manages ROS activity. The image was reproduced with permission [77]

Graphene and its derivatives have gained considerable interest due to their exceptional characteristics, including high surface area, thermal, electrical, and mechanical stability, thereby making them easily used in various applications, including biomedical applications. Moreover, high biocompatibility, mechanical strength, fluorescence, and the ability to penetrate cells makes suitable candidates for biomedical applications, including wound dressing. Additionally, graphene and its derivatives have induced the angiogenesis process by generating reactive oxygen species (ROS) and reactive nitrogen species (RNS). It is important to mention that graphene and its derivatives not only induce the angiogenesis process of wounds but also killed/inhibit the bacterial infections. Indeed, numerous properties of graphene and its derivatives, especially mechanical properties, provide advantages for wound dressing materials [75–77]. Numerous graphene-and their derivatives-based wound dressing materials have been developed and effectively speed up the healing process as well as control bacterial infection at the site of the wound. For instance, Tang et al. synthesised a polydopamine-rGO-chitosan-silk fibroin (PGO-CSF) based scaffold for wound healing. The data indicate that the incorporation of rGO by dual crosslinking of chitosan and silk fibroin notably improved the mechanical properties, including the flexibility of the scaffold. Moreover, the developed scaffold considerably improved the regeneration process of the wound by increasing the promoting factors and decreasing the excessive ROS [77]. Figure 2. A schematic illustration of the synthesis of PGO-CSF-based scaffold for wound healing. Another study focused on the functionalized GO-chitosan (FGOC) hydrogel-based wound dressing materials. The data indicate that the incorporation of GO within the hydrogel significantly improved the compressive stress from 1.9 to 4.2 MPa, whereas tensile stress and strain were improved from 141.2 to 300.2 kPa. The exceptional biocompatibility might improve the adhesion and proliferation of the cells [78]. Najafabadi et al. synthesised the modified GO-chitosan and encapsulated PU foam (GC-PU) for wound dressing materials. The data indicate that the incorporation of GO noticeably improved the thermo-mechanical properties of the GC-PU-based wound dressing materials. Moreover, GO significantly improved the exposure of the cells, which causes inhibition of bacterial growth [79]. Huang et al. fabricated a GO-cyclodextrin-nitric oxide (NO) release (GOC-NO) based hydrogel for wound healing. The data suggested that GO substantially improved the storage modulus with ~ 100 cycles of recoverability by applying 60% strains. Moreover, the release of NO augments the antibacterial ability of the wound dressing materials. Additionally, the synthesised GOC-NO-based wound dressing materials promote angiogenesis and inflammation [80]. Chen et al. fabricated poly-hexamethylene guanidine (PHMG)-GO incorporated into PVA-chitosan (PHMG-GO-PC)-based wound dressing materials. The data indicate that the Young’s modulus increases from 29.29 to 46.32 MPa, whereas elongation at break increases from 216 to 250%. Moreover, high biocompatibility and improved antibacterial activity upon incorporation of GO [81]. The above-mentioned data and Table 1 suggest that graphene and GO exhibit exceptionally high tensile strength and Young’s modulus, enabling them to act as effective nano-reinforcement agents within the polymeric matrix. Indeed, such incorporation within the polymer visibly increased the tensile strength, flexibility, and elasticity, which positively improved the wound healing rate. Additionally, graphene and its derivatives significantly improved durability, which is essential for long-term applications. In general, graphene and its derivatives demonstrate mechanically superior and tunable characteristics, making them highly promising candidates for smart wound dressing applications.

### g-C_3_N_4_-based wound dressing

The g-C_3_N_4_-based biomaterials have attracted significant attention as advanced wound dressing materials not only due to their photo-responsive and antimicrobial activity, but also their tunable mechanical strength while integrating with the polymeric matrix. Indeed, in wound healing applications, mechanical characteristics like tensile strength, flexibility, elasticity, and adhesion to the surface of the wound are of utmost importance for maintaining wound dressing ability [82–84]. Although g-C_3_N_4_ is inherently brittle in nature, encapsulation with polymeric biomaterials such as PVA, chitosan, alginate, and cellulose, etc., considerably improves the mechanical stability of the polymeric composite via strong interfacial interactions, hydrogen bonding, and uniform stress distribution within the polymeric composite. Moreover, the flexibility of wound dressing materials is essential for comfortable contact, thereby allowing them to be easily wrapped around any part of the body [85–87]. In this context, g-C_3_N_4_-based polymeric composites make an exceptional candidate for next-generation smart wound dressing materials.

**Fig. 3:**
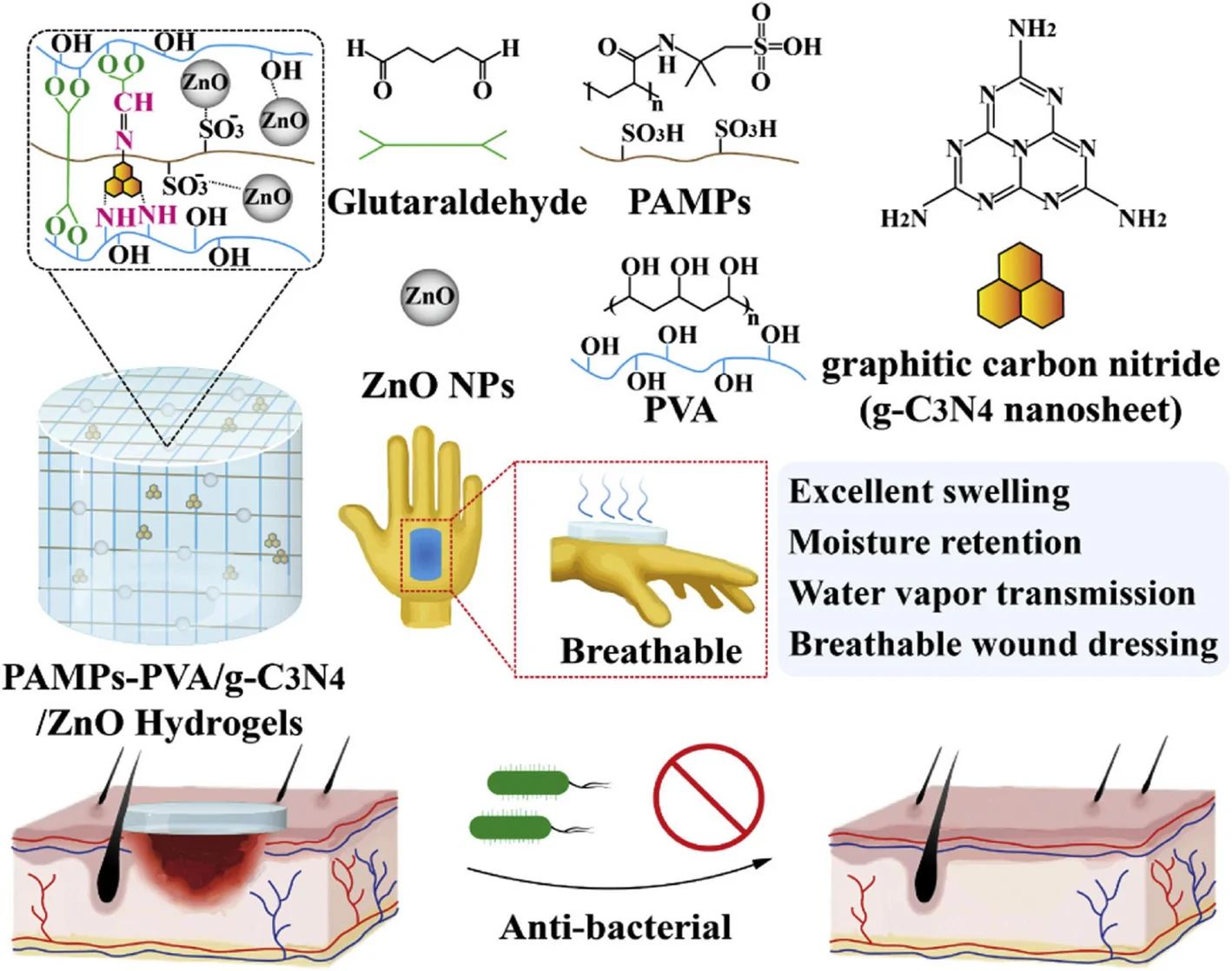
A schematic illustration of the fabricated CZPP-based hydrogel membrane for wound healing application. The figure was reproduced with permission [88]

Numerous g-C_3_N_4_-based polymeric composites have been used as smart wound dressing materials. For instance, Liu et al. fabricated a g-C_3_N_4_-ZnO-polysulfonates-PVA (CZPP)-based hydrogel membrane for wound healing. Figure 3 shows the schematic illustration of the fabricated CZPP-based hydrogel membrane for wound healing applications, which exhibits exceptional air permeability and antibacterial ability, thereby being effectively used for wound healing applications. The prepared CZPP-based hydrogel membrane exhibits mechanical strength (~ 42.6 MPa) and strain (~ 183.35%). Moreover, as-prepared membranes achieved a high swelling ratio (~ 116.06%), moisture retention (~ 90%), cell viability (~ 84.37%), and wound healing rate (88.26%) within 9 days, which was attributed to the CZPP-based hydrogel membrane effectively healing wounds due to exceptional mechanical ability and biocompatibility [88]. Khamene et al. fabricated a g-C_3_N_4_-Ca-aloe vera-PVA-bacterial cellulose (CCAPB)-based wound dressing. The fabricated CCAPB-based wound dressing exhibits high tensile strength (61% elongation) and biodegradation (7% in 96 h). Moreover, the CCAPB-based wound dressing shows an excellent wound healing process, ~ 95% (healing rate) within 21 days of exposure [89]. Youshi et al. fabricated a Myristica fragrans-coated carboxymethyl cellulose-ZnO-g-C_3_N_4_ (M-CZC)-based wound dressing that shows a high wound healing process by controlling bacterial growth, edema, and increasing angiogenesis, collagen synthesis, and epithelialization [90]. Sorourian et al. fabricated BC-Ag-g-C_3_N_4_-Eucalyptus (BACE)-based wound dressing materials. The BACE shows high tensile strength (6.99 MPa) and Young’s modulus (121.37 MPa), which were significantly increased upon incorporation of g-C_3_N_4_. The as-prepared BACE-based wound dressing materials show high swelling behavior, mechanical strength, high cell viability, high antibacterial ability, and visibly reduce the wound area compared with that of the control [91]. Ghazanfaripanah et al. fabricated chitosan-polydopamine-g-C_3_N_4_ with different amounts of g-C_3_N_4_ (CPC)-based hydrogel as a wound dressing material. The as-prepared CPC-based hydrogel showed exceptional mechanical strength from 3.43 kPa to 12.56 kPa upon incorporation of g-C_3_N_4_ within the polymeric matrix. Moreover, upon increasing the amount of g-C_3_N_4_ within the CPC-based hydrogel, the moisture rate decreased significantly due to an increase in molecular density. The CPC-with 10% of g-C_3_N_4,_ shows the exceptional performance in clinical settings by facilitating the wound healing process [92]. Pan et al. fabricated an Ag-PVA-g-C_3_N_4_-(APC) based antibacterial photocatalytic hydrogel for wound dressing. The as-prepared APC-based photo-antibacterial hydrogel has excellent photo-antibacterial activity, mechanical properties, and efficient wound healing [93]. The above-mentioned data and Table 1 suggest that the g-C_3_N_4_ positively increased the mechanical stability of the polymeric matrix, enabling better stress distribution and interfacial interactions. It is important to mention that g-C_3_N_4_-based CB-2DMs tend to be relatively brittle and exhibit lower mechanical strength, thereby being incorporated into polymeric matrices to enhance flexibility. In general, g-C_3_N_4_-based wound dressing materials provide a balance of stiffness and functionality, making them mechanically stable smart wound dressing materials.

### MXenes (carbide)-based wound dressing

**Fig. 4:**
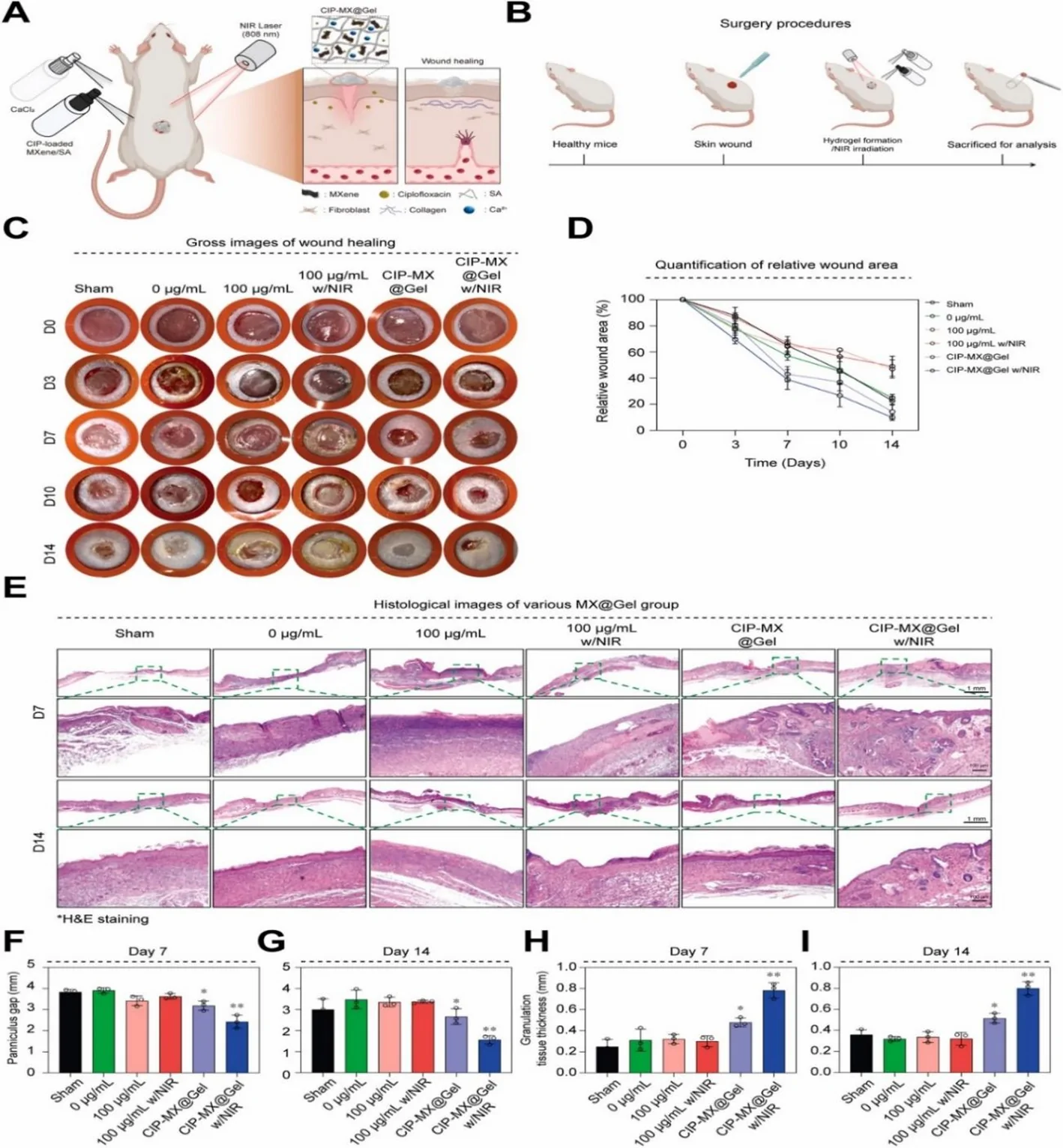
**A** A schematic illustration synthesis and drug release behavior of hydrogel under NIR irradiation, **B** In-vivo healing process, **C** photographic images of wound at different days of exposure, **D** graph of relatively wound area, **E** staining images by using Hematoxylin and eosin at 7 & 14-days exposure, **F**-**G** graph of panniculus gap at 7& 14 days of exposure, and (**H**-**I**). Graph of granular tissue thickness at 7 & 14 days of exposure. The image was reproduced with permission under the Creative Commons license [103]

MXenes are 2D crystalline solids with a considerable diameter range. The unique characteristics of MXenes, like tensile strength, catalytic, magnetic, and electronic properties, attracted significant interest from researchers for end applications. Moreover, tunable surface chemistry of the MXenes might improve their applicability towards end applications. Undoubtedly, MXenes-based materials have become one of the most promising materials in wound dressing applications. MXenes-based wound dressing has emerged as a next-generation material for wound applications due to its exceptional mechanical strength, antibacterial activity, and easily tunable surface chemistry. Indeed, MXenes possess intrinsically high tensile strength and layered structure, which enables efficient stress transfer upon encapsulation with polymers, thereby improving the mechanical integrity of the polymeric matrix by forming interfacial interactions, including electrostatic attractions and hydrogen bonding. Like other CB-2DMs, the enhancement of tensile strength, elasticity, and flexibility is required for skin conformability. Furthermore, incorporating MXenes with polymers significantly improved structural stability that prevents excessive swelling/mechanical collapse of the wound dressing materials. Therefore, MXene-based wound dressing provides an advanced class of multifunctional wound dressing materials for wound care systems [94–100]. Few studies have been done so far, which focus on MXenes-based polymeric composites that have been used as smart wound dressing materials. For instance, Elbialy et al. fabricated chitosan-Ag-Mxenes (CAM)-based polymeric composite as wound dressing materials. The encapsulation of Ag-MXenes with chitosan exceptionally enhances mechanical strength, as elongation breaks increase from 5.3% to 13%. Moreover, CAM-based wound dressing materials show excellent antimicrobial activity, improving free radical scavenging activity, and ~ 29% wound closure compared with chitosan. Furthermore, CAM-based wound dressing materials improved granular tissue, collagen deposition, and α-SMA expression. Indeed, CAM-based wound dressing materials show synergetic effects or combined effects of chitosan, Ag, and MXenes, thereby exhibiting exceptional anti-inflammatory, antioxidant, and antibacterial activity, simultaneously closing the wound. As observed from the data, upon incorporation of Ag-MXenes with chitosan, wound closure was profoundly improved [101]. Cheng et al. fabricated Zn-MOF-BC-MXenes (ZMBM)-based photothermal hydrogel as smart wound dressing materials. The antibacterial activity of ZMBM-based wound dressing materials is due to Zn-MXenes, whereas ZMBM improves wound healing rate by improving the blood vessel regeneration and managing inflammatory responses. Figure 4 shows the synthesis and drug release behavior under NIR irradiation, and the in vivo healing process. As observed from the figure, wound closure significantly improved from 61.03 to 90.14% within 7 to 14 days of exposure, which suggests hydrogels have exceptional tissue regeneration ability [102]. Park et al. fabricated ciprofloxacin loaded-sodium alginate-MXenes (CSAM)-based hydrogel as wound dressing materials. The as-prepared CSAM-based hydrogel exhibits exceptional photothermal ability under NIR irradiation and high biocompatibility. Moreover, the controlled release of the drug based on NIR irradiation, which remarkably improved antibacterial activity. Furthermore, the CSAM-based hydrogel appreciably enhanced wound healing [103]. Li et al. fabricated polydopamine-MXenes-chitosan nonwoven fabric (PMCNF)-based wound dressing materials. The as-prepared PMCNF-based wound dressing materials exhibit excellent wettability, excellent antibacterial activity, blood-clotting ability, and increased fibrinogen reformation, thereby significantly increasing the healing process [104]. The above-mentioned studies and Table 1 suggested that, like other CB-2DMs, MXenes also increased the mechanical stability of the polymeric matrix and wound healing ability. In general, MXenes-based wound dressing materials provide a newer, mechanically stable platform for the polymeric matrix, which makes them next-generation wound dressing materials.

**Table 1:** Different CB-2DMs-based wound dressing materials and their mechanical properties

S. No.	Dressing materials	Mechanical properties	Remarks	Ref
1.	PGO-CSF	Improved flexibility and recoverability up to 4 cycles	rGO significantly improved the mechanical properties as well as accelerated the healing process.	[77]
2.	FGOC	Improved compressive stress from 1.9 to 4.2 MPa, whereas tensile stress and strain improved from 141.2 to 300.2 kPa.	Hydrogel deeply improves cell adhesion and proliferation.	[78]
3.	GC-PU	Improved thermo-mechanical properties of the dressing materials	Improved antibacterial activity due to higher exposure of GO sheets.	[79]
4.	GOC-NO	Improved storage modulus and ~ 100 cycles of recoverability.	Release of NO considerably improved the antibacterial activity.	[80]
5.	PHMG-GO-PC	Young’s modulus increased from 29.29 to 46.32 MPa and elongation at break from 216 to 250%.		[81]
6.	CZPP	CZPP-based hydrogel membrane exhibits mechanical strength (~ 42.6 MPa) and strain (~ 183.35%).	The as-prepared CZPP-based hydrogel membrane exhibits exceptional moisture content, swelling ability, high biocompatibility, and around 89% wound healing rate within 9 days of exposure.	[88]
7.	CCAPB	The CCAPB-based wound dressing exhibits high tensile strength upon incorporation of g-C_3_N_4_.	The CCAPB-based wound dressing shows high healing rates, high antibacterial ability, and biocompatibility.	[89]
8.	M-CZC	**-**	The M-CZC-based wound dressing positively improves the wound healing rate by decreasing edema, bacterial growth, and increasing angiogenesis.	[90]
9.	BACE	The BACE shows high tensile strength (6.99 MPa) and young modulus (121.37 MPa) upon incorporation of g-C_3_N_4_.	The as-prepared BACE shows high biocompatibility, mechanical strength, and a significant reduction of wound area.	[91]
10.	CPC	CPC-hydrogel with 20% of g-C_3_N_4_ shows higher tensile strength (~ 27.2%). The mechanical strength depends upon the amount of g-C_3_N_4_ within the hydrogel.	CPC-based hydrogel improved wound healing by controlling the bacterial infection, reducing inflammation, and influencing gene expression.	[92]
11.	APC	High mechanical strength upon incorporation of g-C_3_N_4_.	The APC-based photo-antibacterial hydrogel is effectively used to treat the healing of wounds.	[93]
12.	CAM	The encapsulation of Ag-MXenes with chitosan significantly enhances mechanical strength, as elongation breaks increase from 5.3 to 13%.	CAM-based wound dressing materials show synergetic effects, thereby exhibiting exceptional anti-inflammatory, antioxidant, and antibacterial activity, simultaneously closing the wound.	[101]
13.	ZMBM	-	ZMBM improves wound healing rate due to the synergetic effects of Zn-MXenes.	[102]
14.	CSAM	-	CSAM-based hydrogel shows the controlled release of the drug based on NIR irradiation and notably enhances wound healing.	[103]
15.	PMCNF	-	PMCNF significantly improved the wound healing process upon incorporation of MXenes.	[104]

The CB-2DMs-based wound dressing materials suggest that graphene, GO, rGO, g-C_3_N_4_, and MXene provide mechanical reinforcement through structural and interfacial mechanisms. CB-2DMs significantly enhance the tensile strength, flexibility, and elastic stability by producing interconnected CB-2DMs reinforcing networks and facilitating efficient stress transfer within the polymeric matrix. However, direct comparison of mechanical values across different studies is quite difficult, as reported characteristics vary with matrix composition, CB-2DMs loading, and testing conditions. Interestingly, higher mechanical strength does not necessarily correspond to superior wound dressing ability. Moreover, excessive incorporation of CB-2DMs might cause aggregation, reduce flexibility, and have adverse effects on cellular compatibility.

Indeed, CB-2DMs have shown promising therapeutic potential; long-term biosafety remains the greatest challenge for clinical translation. It is important to note that numerous factors, such as dose, size, surface chemistry, and exposure time, influence cellular toxicity. Undoubtedly, surface functionalization might improve biocompatibility by controlling the release behaviour; incomplete degradation may lead to long-term tissue accumulation and potential inflammatory and oxidative stress responses. Comprehensive long-term in vivo studies and safety evaluation are essential to establish their clinical safety and translational potential. Therefore, the most promising CB-2DMs-based dressing materials are those that achieve an optimised balance between mechanical integrity and biological functionality rather than maximising a single mechanical parameter.

## Conclusion

The CB-2DMs represent a flexible and highly effective approach for enhancing the mechanical characteristics of wound dressing materials as well as the healing rate. CB-2DMs such as graphene, GO, g-C_3_N_4_, and MXenes offer exceptional reinforcement ability upon incorporation with polymers, thereby improving the mechanical strength and durability of the wound dressing materials. Undoubtedly, the mechanical strength of the wound dressing materials remarkably influences clinical effectiveness by ensuring enhanced adhesion to the wound surface, resistance to deformation, and stability in different physiological conditions. Moreover, with the help of CB-2DMs, we can easily tune the mechanical strength, which might be beneficial for designing according to wound types, such as acute to chronic wounds. However, uniform dispersion, brittleness, and biocompatibility are among the greatest challenges. In this aspect, future research should focus on developing scalable strategies to develop wound dressing materials that have multifunctional properties with minimal mechanical strength, thereby easily achieving high-performance wound dressing materials. The different CB-2DMs exhibit distinct biological functions and therapeutic advantages. Despite their promising wound-healing potential, challenges such as cytotoxicity, biodegradation, scalability, and clinical translation vary among these materials, emphasising the need for application-specific selection of materials. The CB-2DMs have the potential to revolutionise the wound care system by bridging the gap between wound dressing materials, mechanics, and the regenerative wound healing process.

## Data Availability

No datasets were generated or analysed during the current study.
